# Response of Weeping Lantana (*Lantana montevidensis*) to Compost-Based Growing Media and Electrical Conductivity Level in Soilless Culture: First Evidence

**DOI:** 10.3390/plants7020024

**Published:** 2018-03-22

**Authors:** Giuseppe Cristiano, Gjok Vuksani, Vincenzo Tufarelli, Barbara De Lucia

**Affiliations:** 1 Department of Agricultural and Environmental Sciences, University of Bari Aldo Moro, via Amendola 165/A, 70126 Bari, Italy; giuseppe.cristiano@uniba.it; 2 Department of Horticulture and Landscape Architecture, Agricultural University of Tirana, Koder Kamez, 1029 Tirane, Albania; gjvuksani@ubt.edu.al; 3 Department of DETO, Section of Veterinary Science and Animal Production, University of Bari Aldo Moro, 70010 Valenzano, BA, Italy; vincenzo.tufarelli@uniba.it

**Keywords:** organic substrate, pot cultivation, sewage sludge, waste recycling, weeping lantana

## Abstract

The most common substrate for potted ornamental plants is prepared with *Sphagnum* peat; however, the cost and declining availability of high-quality peat, due to environmental constraints, make it necessary to investigate for alternative organic materials. The present study aimed to determine the effects of partial compost replacement with peat and the optimum electrical conductivity (EC) level of the nutrient solution in potted weeping lantana [*L. montevidensis* (Spreng.) Briq.] under a recirculating soilless system. Three compost-based substrates were prepared by mixing peat (Pe) with sewage sludge-based compost (Co.) at a rate of 0% (Pe90Co0Pu10, control), 30% (Pe60Co30Pu10), or 60% (Pe30Co60Pu10), respectively. The soilless recirculated closed system was equipped with two different EC levels (high and low) of nutrient solution. Growing media main characteristics and plant bio-morphometric parameters were evaluated. Our first evidence clearly demonstrates that the replacement of peat with compost at doses of 30% and 60% gave the poorest results for plant diameter, shoots, leaves, flowers, and fresh and dry mass, probably indicating that the physical characteristics of the compost based substrates may be the major factor governing plant growth rate. Compost media pH and EC values, too, showed negative effects on plant growth. Considering the effect of EC level, all morphological traits were significantly improved by high EC compared to low EC in weeping lantana. Thus, based on first evidence, further research is needed on organic materials for the establishment of ecological substrates with optimal physicochemical characteristics for the growth of weeping lantana.

## 1. Introduction

Ornamental greenhouse production has increased in the last decades and is an intensive form of agriculture. Therefore, great amounts of both organic and mineral growing media, mineral fertilizers, water and pesticides are needed to obtain high-quality product in a short cultivation cycle [1].

Rapid growth and flowering in potted plants are mainly correlated to nutrient availability both in the growing media and in fertilizer solutions [2,3]. In Mediterranean floriculture, *Sphagnum* peat is the most representative component in the growing media preparation; however, environmental concerns [4] as well as increasing prices have generated significant interest in the development of alternatives to peat [5]. Previous studies have investigated peat replacement with organic wastes originating from composting [6,7]. The compost is principally produced by urban and agro-industrial sludge and can be reused in soil as nutrient-rich fertilizers [8] and also as soilless growing media, growing seedlings, plant propagation and ornamental pot production [9]. Moreover, the proportion of compost in the final substrate is very important in order to minimize potential hazards, especially high salinity and heavy metal contents [10].

Soilless culture is considered one of the main components of sustainable horticulture. In the open soilless ornamental pot cultivation, a surplus of nutrients and water is allowed to run to waste; thus, to achieve better efficiency, save irrigation water and to reduce groundwater contamination in order to obtain a sustainable production process, plants should be cultivated under a closed soilless system with a recirculating nutrient solution [11]. In the Mediterranean basin, in soilless ornamental pot cultivation, the most widely used is the drip irrigation system, where salts not taken up by the crop accumulate in the recirculating nutrient solution, which has to be flushed out frequently [12]. Electrical conductivity of nutrient solution is one of the most important factors influencing the success of the soilless system [13,14]. In potted geranium production, the nutrient application rate was reduced by 50% without any detrimental effect on plant growth and quality; in fact, some ornamentals are characterized by low nutrient uptake [15].

The Lantana genus (*Verbenaceae* family) is composed by 150 herb and shrub species, mainly native to tropical America, with several species native to Africa and Asia [16]. The weeping lantana [*L. montevidensis* (Spreng.) Briq.], a low woody shrub with horizontally growing branches, is native to tropical areas of South America and it is becoming a popular groundcover and landscape plant in frost-free areas. The weeping lantana has dark green leaves with a rough hairy texture and is a continuous bloomer, producing quantities of small flowers that are arranged in flower head clusters [17]. Based on our literature review, no experimental trials have yet been conducted to correlate the compost dose in peat substitutions with the concentration of nutritive solution in weeping lantana. Therefore, based on these considerations, the objective of the present study was to determine the effects of the compost dose and the nutrient solution concentration in potted creeping weeping lantana under a recirculating soilless system.

## 2. Results

The growing media proposed were found to differ significantly in terms of the main physicochemical characteristics at transplant (Table 1). The pH values of all mixtures were around pH 6.38, ranging within pH 5.84–6.79, except for the mixture Pe90Co0Pu10, whose pH was significantly lower than that of Pe60Co30Pu10 and Pe30Co60Pu10. Electrical conductivity (EC) was increased by the addition of both composts (on average +53% in Pe60Co30Pu10 and +100% Pe30C60Pu10, respectively) compared with the peat control. 

The concentrations of nutrient ions (total N, P, and K) were found to be enhanced when compost was used to replace peat volume. pH was significantly higher in the SC mixtures only when it was used to replace peat volume at a rate of 50%. Among the physical parameters, dry bulk density was significantly higher in both composts than in the control, while an opposite trend was found for the total pore space and water holding capacity (Table 1). 

Table 2 reports the effects of the tested growing media and EC on plant diameter.

The mixture affected the weeping lantana diameter, which was significantly reduced compared with the peat control, although no significant differences were found when comparing the effects of the two doses of compost applied. Considering the effect of EC level, the plant diameter was significantly improved by high EC compared to low EC (at 145 and 180 DAT). 

Applying compost to weeping lantana significantly decreased the number of shoots (Table 3), leaves (Table 4), and flower (Table 5), in comparison with the peat control. The same trend was also observed when evaluating the plant leaf area (Table 6). However, considering all of these parameters, it was found that a high EC level positively influenced the weeping lantana traits compared to a low EC level. In particular, with regard to number of flowers, the two mixtures containing compost performed at the same level. Furthermore, the interaction effect between the compost dose and the EC level turned out to be significant for the analyzed plant traits at most of the considered DAT.

Finally, the effects of the compost dose and EC level on plant fresh and dry mass at different DAT are reported in Table 7 and Table 8, respectively. The two composts significantly affected the weeping lantana fresh and dry mass, which was reduced compared with the peat control. Overall, the two mixtures containing compost performed at the same level with regard to fresh and dry mass.

## 3. Discussion

Previous studies evaluating the effects of compost-based growing media and electrical conductivity in potted creeping weeping lantana under a recirculating soilless system have not yet been reported. Thus, cross-referencing in the discussion of the findings in this study will be based on available results from other plant species of ornamental interest or not. 

Much research has focused on the beneficial effects on plant growth of moderate (up to 30%) compost doses into mixtures [9,10]; therefore, based on these important environmental benefits, one of the major concerns in its operative use is the standardization of compost characteristics [19].

The suitability of compost for floriculture application mainly depends on physical and chemical parameters. The increase in pH in the present study, caused by the addition of compost in the growing media, was also found by Heiskanen [20], Ostos et al. [21] and Massa et al. [22], with negative consequences on plant performance. 

In our study, both control and Pe60Co30Pu10 mixtures pH values were within the established limits for an ideal substrate [18]. According to Abad et al. [18], an acceptable EC value should be lower than 0.5 dSm^−1^; and in our trial the Pe60Co30Pu10 and Pe30Co60Pu10 EC values were strongly affected by the addition of compost. As a consequence, there values should be harmful for lantana growth, as it is among the crops that are moderately tolerant to salinity [23,24]. In the salt-sensitive calceolaria hybrid, electrical conductivity and chloride concentration were the main factors influencing plant growth [9]. In container Norway spruce seedlings, the higher EC of the compost medium contributed to their non-germination [20]. 

In soil compost application, the only limiting factor may be the increased soil salinity [25]. In previous studies using compost, it was demonstrated that salinity can be an important limiting factor [26,27]. When compost replaces peat in growing media, it is of great importance that it is sufficiently stabilized in order to avoid the negative growth effects due to N mineralization, oxygen depletion, or the presence of phytotoxic compounds [28]: the compost used reached a good degree of maturity according to its carbon to nitrogen ratio.

In our study, applying compost increased the nutrient content, and this result has been widely reported by many previous studies. Ingelmo et al. [29] showed an increase in nitrogen concentration in soils treated with sewage sludge or municipal soil waste compost, compared with untreated soil [30,31]. Furthermore, an increase in phosphorous and potassium contents was reported, as the compost application rate increased when dairy cattle manure compost was applied. In many cases, the levels of nutrients are so high that they inhibit plant growth by inducing osmotic stress [32].

The marketability of potted lantana is greatly influenced by the conditions of their production. The most important factors are related to substrate quality, drainage, irrigation, water quality, and fertilization [33].

Many studies have focused on the physical properties of compost-based growing media as a growth-limiting factor. Grigatti et al. [7] compared mixtures formed by 25-50-75-100% compost made from green waste and sewage sludge, amended with peat moss, as potting media. The compost increase caused a reduction in the water holding capacity of the media; therefore, the best agronomic performance in the *Begonia* was realized with the addition of only 25% compost. Heiskanen [20] reports that the compost additive in peat changed both the bulk density and the water-holding capacity, thereby decreasing seedling masses.

Plant responses to compost treatments are highly dependent on the species, source, and dose of compost utilized [7]. Growth limitation originates from nutritional imbalances and compost salinity [34]. Our results are in agreement with those reported by Papafotiou et al. [35], who showed a reduction of all agronomical values in *Poinsettia* due to the increasing replacement of peat by olive mill compost. The high EC of media with composted sewage sludge at the transplant may be the reason for the negative effect on weeping lantana plant development, together with the low EAW of the media with 30% and 60% compost that could also have negatively affected plant growth. A similar effect was found in *Ficus benjamina* [36], *L. camara* [37], and *Calceolaria* [9]. 

Weeping lantana plants in our experiment showed high rusticity and adaptability, demonstrating their viability for recycling soilless cultivation. Actually, there is an increasing need to recirculate and reuse nutrient solutions in order to reduce the environmental impact and economic costs [38,39]. However, managing the nutrient solution is one of the biggest challenges in hydroponics due to the limited substrate volume [40]. Maintaining a favorable electrical conductivity in the lower layers of the substrate is crucial for optimal crop performance. The half nutrient solution did not meet the nutrient requirements of lantana plants, as confirmed by Rouphael and Colla [41] in zucchini squash soilless cultivation; therefore, the product quality was obtained using a solution with high electrical conductivity. 

## 4. Materials and Methods 

### 4.1. Growing Media Preparation

Composts, peat, and pumice were tested as growing media. The commercial compost (local waste compost by Eden ’94, Manduria (TA), Italy) utilized (C/N < 12; germination index > 50%) in the mixtures was about four months old and obtained by mixing 70% (*v*:*v*) of olive tree pruning and 30% of sewage sludge (60% food processing and 40% waste water). 

The *Sphagnum* white plus black peat (C/N 40.4) was also a commercial substrate (Plantaflor^®^, Vechta, Germany); pumice was a volcanic rock providing good aeration for plants (Europomice s.r.l., Vechta Putigliano (GR), Italy). At transplantation, a total of 1 L of sample per substratum and replication was collected for laboratory analysis. 

The main physical and chemical properties of growing media were analyzed following the procedures indicated by De Lucia et al. [2], according to the European standards (EN13037 [42], EN13038 [43] and EN13041 [44]). None of the analyzed parameters exceeded the Italian regulations. Three compost-based substrates were prepared by mixing peat (P) with sewage sludge-based compost (C) at rates of 0% (Pe90Co0Pu10, control), 30% (Pe60Co30Pu10), and 60% (Pe30Co60Pu10), respectively, with no previous fertilization. In all the growing media, 10% of the volume was represented by pumice. 

### 4.2. Experimental Procedures

On 6 October 2016, rooted cuttings of weeping lantana [*L. montevidensis* (Spreng.) Briq.] ‘New Gold’ were individually transplanted into brown plastic pots (2.6 L of volume), and filled with growing media. The cultivation was carried out from 8 October 2016 to 5 April 2017 at the Campus Experimental Farm of the Department of Agro-Environmental and Territorial Science (University of Bari Aldo Moro, Bari, Italy; latitude 41°08′ N; longitude 16°51′ E; 16 m a.s.l.), in a plastic greenhouse having a minimum temperature of ≥16/14 °C (day/night), whereas above 22 °C temperature was controlled by natural ventilation. Pots were placed on aluminum benches with a density of nine plants⋅m^−2^ and watered by drip irrigation.

The soilless recirculated closed system, through the recycling of run-off, was equipped with two different levels of nutrient solution: high (HEC) having pH = 6.5, EC = 2.2 dSm^−1^, mineral content (ppm) of 172 N, 31.7 P, 252.6 K, 33.2 Mg, 87.2 Ca, 11.5 S, 1.6 Fe, 0.3 Zn, 0.3 B, 08 Cu; and low (LEC) having pH = 6.6, EC = 1.1 dSm^−1^ obtained from HNS (full) as half strength. Every bench was served by a tank containing 50 L of nutrient solution. Every seven days fresh nutrient solution was added to the tanks, up to the initial volume of 50 L, in order to replace the nutrient solution consumed.

In both nutrient solutions the pH and EC were monitored weekly and measured using a portable pH meter (HI 9025) and a conductivity meter (HI-9033) (Hanna Instruments). The number of fertigations per day was two (October–November), one (December–February), or three (March–April). Fertigation was set up in order to obtain about 40% of drainage. All pots received a total of 140 mL day^−1^ per fertigation during the whole experimental period. Both nutrient solutions were sterilized with a sand filter to avoid the development of disease. The experimental treatments started on the day of planting.

Both in vivo and destructive evaluations of plant diameter, number of shoot, leaves and flowers, biomass accumulation (as fresh and dry weight of plants), and total leaf area were carried out. Plant fresh weight (FW) was measured by an analytical balance (0.001 decimal places). Individual plant parts were dried in an oven at 70 °C for 48 h to determine the dry weight (DW). Total leaf area was assessed using a LI-COR 3100 area meter. The plant growth was determined 90, 120, and 150 days after transplant (DAT). On each sampling date three plants per treatment were randomly harvested and used for the biometric measurements. At the end of the trial, 162 plants (54 plants/block) were harvested. Furthermore, a sample of leachate was taken for each treatment weekly to evaluate the pH and EC values.

### 4.3. Statistical Analysis

The three compost doses were combined with two different salinity levels in a two-factorial experimental design. The treatments were arranged in a randomized complete-block design with three replicates per treatment, amounting to a total of 18 experimental unit plots with nine plants each. Analysis of variance (ANOVA) of the experimental data was performed using CoStat—Statistics Software (Monterey, CA, USA). The significance level of results was tested by ANOVA (*F*-test) at *p* ≤ 0.05 and the treatment’s means were compared by a Student Newman–Keuls (SNK) test at *p* ≤ 0.05.

## 5. Conclusions

The present findings must be considered preliminary; however, they provide interesting evidence that clearly demonstrates that the replacement of peat with compost at doses of 30% and 60% gave the poorest results for weeping lantana diameter, shoots, leaves, flowers, and fresh and dry mass, probably indicating that the physical characteristics of the compost-based substrates may be the major factor governing the plant growth rate. Compost media pH and EC values, too, showed negative effects on plant growth.

Considering the effect of the EC level of nutrient solutions, all morphological traits were significantly improved by high EC compared to low EC. Thus, based on this first evidence, further research is needed on organic materials for the establishment of ecological substrates with optimal physical–chemical characteristics for the growth of weeping lantana.

## Figures and Tables

**Table 1:** Main physical and chemical characteristics of growing media at transplant, measured on three replicates.

Parameters	Compost-Based Growing Media			
	Pe90Co0Pu10	Pe60Co30Pu10	Pe30Co60Pu10	Critical Value 0.05
pH (1:10)	5.84b	6.51a	6.79a	0.29
EC (dSm^−1^)	1.10c	1.68b	2.21a	0.26
Total N (mg kg^−1^)	380c	417b	572a	19.1
P (mg kg^−1^)	190c	1390b	1809a	18.2
K (mg kg^−1^)	370c	2289b	2728a	10.1
DBD (g cm^−3^) ^(1)^	245c	370b	445a	21.9
TPS (% *v*/*v*) ^(2)^	94.8a	88.2b	84.3c	1.46
WHC pF1 (%) ^(3)^	80.0a	72.0b	65.0c	5.60

In each row, different letters indicate significant differences for *p* ≤ 0.05 (SNK test). ^(1)^ Optimum value: <400 g cm^−3^; ^(2)^ optimum value: >85% *v*/*v*; ^(3)^ optimum value: 55–70% *v*/*v* [18]; Dry bulk density: DBD; Total pore space: TPS; Water holding capacity: WHC.

**Table 2:** Effect of compost dose and EC level on plant diameter (cm) on 110, 145 and 180 days after transplant (DAT).

Treatments	Days after Transplant (DAT)		
	110	145	180
*Compost dose (C)*			
Pe90Co0Pu10	76.8a	103.5a	139.8a
Pe60Co30Pu10	62.8b	92.1c	116.2b
Pe30Co60Pu10	51.2c	98.1b	117.5b
Critical value 0.05	1.44	3.95	5.91
*EC level (EC)*			
LEC	61.9b	98.3b	117.7b
HEC	65.3a	104.2a	131.3a
Critical value 0.05	1.17	3.23	4.83
*Significance*			
C	**	*	**
EC	**	**	**
C × EC	**	**	NS

Different letters within each column indicate significant differences according to SNK test at *p* ≤ 0.05. NS not-significant; *p < 0.05; ** *p* < 0.01.

**Table 3:** Effect of compost dose and EC level on plant shoots (n) on 110, 145 and 180 days after transplant (DAT).

Treatments	Days after Transplant (DAT)		
	110	145	180
*Compost dose (C)*			
Pe90Co0Pu10	35.7a	82.7a	124.8a
Pe60Co30Pu10	29.5b	40.7b	85.2b
Pe30Co60Pu10	23.0c	42.8c	74.5c
Critical value 0.05	2.94	4.44	9.78
*EC level (EC)*			
LEC	25.9b	52.3b	86.4b
HEC	30.9a	61.4a	103.2a
Critical value 0.05	2.40	3.62	7.99
*Significance*			
C	**	**	**
EC	**	**	**
C × EC	NS	*	**

Different letters within each column indicate significant differences according to SNK test at *p* ≤ 0.05. NS not-significant; * *p* < 0.05; ** *p* < 0.01.

**Table 4:** Effect of compost dose and EC level on plant leaves (n) on 110, 145 and 180 days after transplant (DAT).

Treatments	Days after Transplant (DAT)		
	110	145	180
*Compost dose (C)*			
Pe90Co0Pu10	309.7a	856.8a	1147.3a
Pe60Co30Pu10	226.8b	425.2b	911.8b
Pe30Co60Pu10	214.0c	386.2c	768.2c
Critical value 0.05	10.25	13.98	26.90
*EC level (EC)*			
LEC	232.2b	524.0b	918.9b
HEC	268.1a	588.1a	1099.3a
Critical value 0.05	8.37	13.98	21.97
*Significance*			
C	**	**	**
EC	**	**	**
C × EC	*	**	**

Different letters within each column indicate significant differences according to SNK test at *p* ≤ 0.05. NS not-significant; * *p* < 0.05; ** *p* < 0.01.

**Table 5:** Effect of compost dose and EC level on plant flowers (n) on 110, 145 and 180 days after transplant (DAT).

Treatments	Days after Transplant (DAT)		
	110	145	180
*Compost dose (C)*			
Pe90Co0Pu10	13a	18a	148a
Pe60Co30Pu10	3b	5b	42b
Pe30Co60Pu10	4b	5b	42b
Critical value 0.05	0.08	1.38	8.36
*EC level (EC)*			
LEC	4b	6a	62b
HEC	10a	8a	94a
Critical value 0.05	1.43	2.12	6.82
*Significance*			
C	**	**	**
EC	**	NS	**
C × EC	**	**	**

Different letters within each column indicate significant differences according to SNK test at *p* ≤ 0.05. NS not-significant; * *p* < 0.05; ** *p* < 0.01.

**Table 6:** Effect of compost dose and EC level on specific leaf area (cm^2^).

Treatments	Days after Transplant (DAT)		
	110	145	180
*Compost dose (C)*			
Pe90Co0Pu10	2.69a	2.86a	2.99a
Pe60Co30Pu10	2.24b	2.31b	2.47b
Pe30Co60Pu10	1.88c	2.03c	2.11c
Critical value 0.05	0.10	0.24	0.13
*EC level (EC)*			
LEC	2.26a	2.80b	2.69b
HEC	2.28a	3.41a	3.30a
Critical value 0.05	0.08	0.19	0.11
*Significance*			
C	**	**	**
EC	NS	**	**
C × EC	*	**	**

Different letters within each column indicate significant differences according to SNK test at *p* ≤ 0.05. NS not-significant; * *p* < 0.05; ** *p* < 0.01.

**Table 7:** Effect of compost dose and EC level on plant fresh mass (g) on 110, 145 and 180 days after transplant (DAT).

Treatments	Days after Transplant (DAT)		
	110	145	180
*Compost dose (C)*			
Pe90Co0Pu10	73.0a	191.7a	408.9a
Pe60Co30Pu10	40.6b	115.9b	176.5b
Pe30Co60Pu10	33.0c	114.1b	159.6b
Critical value 0.05	2.56	12.83	19.80
*EC level (EC)*			
LEC	43.4b	137.8a	224.7b
HEC	54.4a	143.4a	272.0a
Critical value 0.05	2.09	10.47	16.19
*Significance*			
C	**	**	**
EC	**	NS	**
C × EC	*	**	*

Different letters within each column indicate significant differences according to SNK test at *p* ≤ 0.05. NS not-significant; * *p* < 0.05; ** *p* < 0.01.

**Table 8:** Effect of compost dose and EC level on plant dry mass (g) on 110, 145 and 180 days after transplant (DAT).

Treatments	Days after Transplant (DAT)		
	110	145	180
*Compost dose (C)*			
Pe90Co0Pu10	15.0a	41.6a	88.4a
Pe60Co30Pu10	7.5b	23.8b	33.5b
Pe30Co60Pu10	6.7b	23.2b	31.6b
Critical value 0.05	1.42	3.58	9.68
*EC level (EC)*			
LEC	9.3a	28.5a	46.2b
HEC	10.2a	30.4a	56.2b
Critical value 0.05	1.16	2.92	7.90
*Significance*			
C	**	**	**
EC	NS	NS	*
C × EC	NS	**	NS

Different letters within each column indicate significant differences according to SNK test at *p* ≤ 0.05. NS not-significant; * *p* < 0.05; ** *p* < 0.01.
